# Regulation of Soluble E-Cadherin Signaling in Non-Small-Cell Lung Cancer Cells by Nicotine, BDNF, and β-Adrenergic Receptor Ligands

**DOI:** 10.3390/biomedicines11092555

**Published:** 2023-09-18

**Authors:** Ravel Ray, Stuti Goel, Hind Al Khashali, Ban Darweesh, Ben Haddad, Caroline Wozniak, Robert Ranzenberger, Jeneen Khalil, Jeffrey Guthrie, Deborah Heyl, Hedeel Guy Evans

**Affiliations:** Chemistry Department, Eastern Michigan University, Ypsilanti, MI 48197, USA; rray9@emich.edu (R.R.); sgoel1@emich.edu (S.G.); halkhash@emich.edu (H.A.K.); bdarwees@emich.edu (B.D.); bhaddad1@emich.edu (B.H.); cwoznia5@emich.edu (C.W.); rranzenb@emich.edu (R.R.); jkhalil2@emich.edu (J.K.); jguthri7@emich.edu (J.G.); dheylcle@emich.edu (D.H.)

**Keywords:** soluble E-cadherin, nicotine, BDNF, β-adrenergic receptors, matrix metalloproteases, lung cancer, nicotinic acetylcholine receptors, p53, PI3K, ERK1/2, EGFR, IGF-1R

## Abstract

The ectodomain of the transmembrane protein E-cadherin can be cleaved and released in a soluble form referred to as soluble E-cadherin, or sE-cad, accounting for decreased E-cadherin levels at the cell surface. Among the proteases implicated in this cleavage are matrix metalloproteases (MMP), including MMP9. Opposite functions have been reported for full-length E-cadherin and sE-cad. In this study, we found increased MMP9 levels in the media of two non-small cell lung cancer (NSCLC) cell lines, A549 and H1299, treated with BDNF, nicotine, or epinephrine that were decreased upon cell treatment with the β-adrenergic receptor blocker propranolol. Increased MMP9 levels correlated with increased sE-cad levels in A549 cell media, and knockdown of MMP9 in A549 cells led to downregulation of sE-cad levels in the media. Previously, we reported that A549 and H1299 cell viability increased with nicotine and/or BDNF treatment and decreased upon treatment with propranolol. In investigating the function of sE-cad, we found that immunodepletion of sE-cad from the media of A549 cells untreated or treated with BDNF, nicotine, or epinephrine reduced activation of EGFR and IGF-1R, decreased PI3K and ERK1/2 activities, increased p53 activation, decreased cell viability, and increased apoptosis, while no effects were found using H1299 cells under all conditions tested.

## 1. Introduction

One of the most frequently diagnosed cancers is lung cancer, which is a leading cause of cancer-related deaths worldwide [1,2]. Non-small cell lung cancer (NSCLC) is considered the most common type of lung cancer and accounts for the majority of all lung cancer diagnoses [3,4].

**Figure 1:**
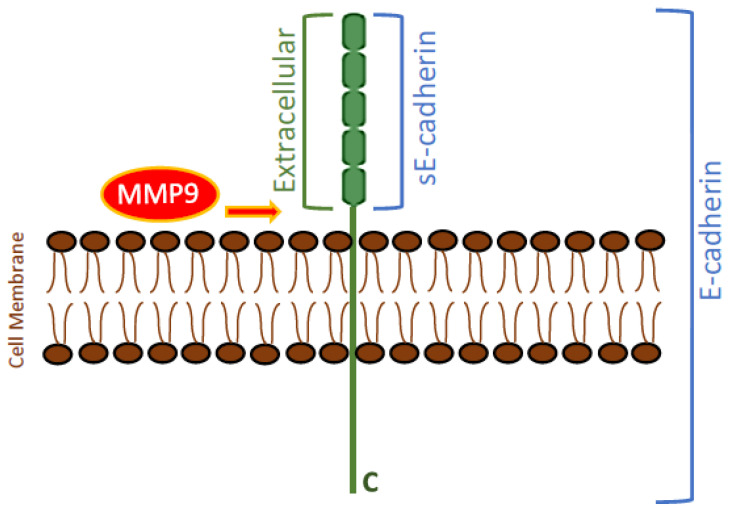
Schematic representation of an E-cadherin molecule and its proteolysis by MMP9. Extracellularly, cleavage of E-cadherin by MMP9 (arrow) generates soluble E-cadherin (sE-cadherin), an extracellular fragment of 80 KDa. C: Cytoplasmic C-terminal domain.

Strong cell-cell interactions are known to be a barrier to the mobility of cancer cells [5,6]. A fundamental change reported during cancer progression to an invasive state is the loss of intercellular adhesion by E-cadherin, a homophilic, calcium-dependent adhesion protein localized to adherens junctions between epithelial cells and known to have tumor suppressor functions [6,7,8]. E-cadherin is a single-pass type-I transmembrane glycoprotein composed of an extracellular domain, a transmembrane segment, and a cytoplasmic domain (Figure 1) [7,8]. The extracellular domain is composed of five extracellular cadherin domain repeats that bind calcium ions, forming a stiff linear molecule (Figure 1) [6,7]. The cytoplasmic domain interacts with the catenins and a number of actin-binding proteins to tether the cadherin-catenin complex to the actin cytoskeleton [6]. 

Ectodomain shedding, a common process that cells use to modify the function of membrane proteins, involves proteolytic processing and liberation of the extracellular domain fragments of membrane proteins [9]. Several studies have shown that this process occurs widely during tumor progression [9]. E-cadherins are known to undergo proteolysis on the extracellular face of the plasma membrane, catalyzed by proteases including matrix metalloproteinase-9 (MMP9), disintegrin and metalloproteinase 10 (ADAM10), plasmin, kallikrein, and other sheddases [7,10,11,12,13]. This proteolytic cleavage converts the mature 120 kilodalton (kDa) E-cadherin into an intracellular C-terminal 38 kDa fragment and an ectodomain N-terminal 80 kDa fragment, referred to as soluble E-cadherin (sE-cad), that is released from the plasma membrane [8,11,12,13]. This 80 kDa fragment contains the five extracellular domains of E-cadherin [8,11,12,13]. The soluble proteolytically cleaved sE-cad form differs in its function from the membrane-tethered E-cad form [6,7,8,14]. While E-cad plays a key role in cellular adhesion, sE-cad disrupts and interferes with the intercellular adherens junctions in part by damaging and disturbing existing ones [6,8,11,12,13]. In addition, the activities of a number of MMPs and ADAMs are known to be increased by sE-cad, and through binding to surface receptors, sE-cad acts as a signaling molecule with oncogenic functions that leads to increased tumor survival, progression, and growth of various types of cancer, including lung cancer [11,12]. 

While the neurotransmitter acetylcholine (ACh) is well-known for mediating synaptic transmission, it has also been shown to act as an autocrine and paracrine growth factor in human lung cancer cells known to express all the proteins of the ACh-signaling system necessary for the synthesis and degradation of ACh [15,16,17,18]. ACh has been reported to accelerate the proliferation of lung cancer cells by binding to nicotinic ACh receptors (nAChRs), which are members of the pentameric ligand-gated ion channels, and to muscarinic ACh receptors that belong to the family of G protein-coupled receptors (GPCRs) [15,18,19,20,21]. ACh and nicotine bind the nAChRs as the endogenous and exogenous ligands, respectively [15,22,23]. Activation of nAChRs plays an important role in cholinergic signaling and the survival, proliferation, progression, and metastasis of a range of cancers, including lung cancer [15,19,22,24,25,26]. 

Expression of β-adrenergic receptors (β-ARs), a class of seven transmembrane GPCRs, has been shown in lung cancer cells and functions to increase cellular proliferation, apoptosis resistance, and metastasis [27]. While binding of nicotine to nAChRs is the principal mode of its biological action, nicotine has also been found to trigger the production of β-AR ligands, for example, adrenaline and noradrenaline, that act via the β-ARs, activating a number of oncogenic and mitogenic signaling pathways, leading to cell proliferation and the development of lung cancer [24,28]. 

Acting via its primary receptor, tropomyosin receptor kinase B (TrkB), brain-derived neurotrophic factor (BDNF), a member of the neurotrophin family of growth factors, is widely known for neuronal survival and the maintenance of synapses [29]. More recently, many reports have elucidated the mechanisms by which neurotrophins operate in different cancers [29,30,31]. BDNF was reported to accelerate the progression of many cancers [32,33], including lung cancer, promoting invasion of lung squamous cell carcinoma [32,33,34]. Upregulation of BDNF levels in lung cancer cell culture supernatants was observed compared with normal lung and linked to lung tumorigenesis and poor prognosis in NSCLC patients [32]. Using NSCLC cell lines, we previously found that the levels of proBDNF were higher in the media of A549 (p53 wild-type) cells than in H1299 (p53-null) cells [35]. We also reported that the amount of amyloid beta was decreased by mBDNF signaling and correlated with the increased release of soluble amyloid precursor protein α from NSCLC cells [36]. More recently, we found an increase in cell viability and cisplatin resistance upon NSCLC cell treatment with nicotine and/or BDNF, effects opposite to those observed by treatment of cells with propranolol [37].

The aim of this study was to investigate the regulation of sE-cad levels in human lung cancer cell media by nicotine, BDNF, and/or β-AR ligands and how sE-cad can promote cell survival and block apoptosis via enhanced epidermal growth factor receptor (EGFR) and insulin-like growth factor 1 receptor (IGF-1R) signaling. 

## 2. Materials and Methods

### 2.1. Materials

Most of the material used in this study was purchased, as we reported earlier [38,39,40,41,42,43]. Phosphate-buffered saline (PBS), nitrocellulose membranes, nicotine, BDNF, epinephrine, and propranolol hydrochloride were purchased from Sigma-Aldrich. The caspase 3 (cleaved) colorimetric In-Cell ELISA Kit (62218), Halt Protease and Phosphatase Inhibitor Cocktail, BCA protein assay kit, super signal west pico luminol (chemiluminescence) reagent, human IgG (hIgG) isotype control, α-tubulin monoclonal antibody (DM1A), goat anti-mouse IgG (H + L) superclonal secondary antibody, HRP conjugate (A28177), 3,3′,5,5′-tetramethylbenzidine (TMB), and lipofectamine 2000 transfection reagent were from ThermoFisher. Donkey anti-mouse IgG (HRP) (ab205724) was purchased from Abcam. MMP9 siRNA (sc-29400), MMP9 antibody (sc-393859), m-IgG Fc BP-HRP: sc-525409, and anti-E-cadherin antibody (DECMA-1, sc-59778) were from Santa Cruz Biotechnology. SignalSilence Control siRNA (unconjugated, 6568) was purchased from (Cell Signaling Technology, Danvers, MA, USA). 

### 2.2. Cell Culture

Human NSCLC cell lines, A549 (ATCC CCL-185) and H1299 (ATCC CRL-5803), were purchased from the American Type Culture Collection (ATCC, Manassas, VA, USA). Cells were cultured as we reported earlier [38,39,40,41,44,45] in DMEM/F12 media containing +10% fetal bovine serum, 50 U/mL penicillin, and 50 U/mL streptomycin at 37 °C, 95% humidity, and 5% CO_2_. The cells were counted with a hemocytometer after trypan blue staining. A549 and H1299 cells were passaged when they achieved 80–90% confluence by trypsinization using standard methods at ~1:5–1:10 dilutions and were cultured no longer than 3 weeks. After thawing, the passage number did not exceed 20. The cells were routinely monitored for morphological changes and p53 status by ELISA and Western blotting. 

### 2.3. MMP9 Level Measurement

The levels of MMP9 in the cell culture media were measured as we reported earlier [35,45] using the Invitrogen human MMP9 solid-phase sandwich ELISA kit. In brief, a matched antibody pair was used to measure the amount of target bound. The signal was proportional to the concentration of the target and detected after the addition of a substrate solution. 

### 2.4. Activated EGFR Assay

Activated (phosphorylated) EGFR was quantitated using the Phospho-EGFR (Y1068) + pan-EGFR ELISA Kit (RAB0166) from Sigma-Aldrich according to the manufacturer’s recommendation. Briefly, samples were added to a 96-well plate precoated with an anti-EGFR antibody that binds both the phosphorylated and pan-EGFR present in the sample. The ratio of phosphorylated EGFR to total EGFR was calculated and plotted for each condition.

### 2.5. Activated IGF-1R Assay

The Phospho-IGF-1R (Tyr1165/1166) and Total IGF-1R ELISA kits (RayBiotech, Peachtree Corners, GA, USA) were used to quantitate activated (phosphorylated) IGF-1R according to the manufacturer’s instructions and as previously reported [37]. Color development was measured in the linear range in proportion to the amount of IGF1R (Tyr1165/1166) or pan IGF1R bound to the wells of a 96-well plate. After addition of the stop solution, the color changed from blue to yellow, and then the color intensity was measured at 450 nm.

### 2.6. Phosphoinositide 3-Kinase (PI3K) Assay

The PI3-kinase p85-alpha/gamma (Phospho-Tyr467/199) colorimetric cell-based lysate-free ELISA kit (Boster, Pleasanton, CA, USA, EKC2337) was used following the manufacturer’s instructions. Primary antibodies targeting total PI3K p85 and phosphorylated-PI3K p85 (recognizes p85 PI3K alpha/gamma phospho-tyrosine 467/199) were used. The relative number of cells in each well was determined using a crystal violet solution. The signals for phospho-PI3K and total-PI3K were each normalized to cell number, and then the ratio of phospho-PI3K to total-PI3K was determined and plotted for each treatment. 

### 2.7. Extracellular Signal-Regulated Kinase (ERK) Assay

The ERK1/2 (pT202/Y204 + Total) ELISA kit (Abcam, Waltham, MA, USA, ab176660) was used to quantitate ERK according to the instructions provided by the manufacturer and our previously published work [36]. The assay uses an antibody mix containing an affinity-tagged capture antibody and a reporter-conjugated detector antibody. The signal was detected by absorbance measurements at 450 nm. The ratio of phospho-ERK1/2 to total ERK1/2 was determined and plotted for each treatment. 

### 2.8. p53 Transcription Factor Activity Assay

The activity of p53 was assayed using the human p53 transcription factor activity assay kit (Catalog #: TFEH-p53) (RayBio, Peachtree Corners, GA, USA), as we reported earlier [35,42,43]. Active p53 contained in whole cell lysates was specifically captured by double-stranded oligonucleotides bound to 96-well plates that contain the p53 binding sequence. 

### 2.9. MTT Assay

Cell (0.2 × 10^5^/well) viability was measured in 96-well plates using the MTT reduction assay (Sigma-Aldrich, Burlington, MA, USA), as we reported previously [38,41,46]. All absorbance measurements (570 nm) were in the linear range. Untreated cells or wells containing only media and DMSO were used as positive and negative controls, respectively. Statistical analysis was conducted using GraphPad Prism version 9.5.1 for Windows. 

### 2.10. Apoptosis Assay

For the caspase 3 (cleaved) colorimetric assay, activated (cleaved) caspase 3 and tubulin were simultaneously measured in triplicate in whole cells by an in-cell ELISA assay (ThermoFisher, Waltham, MA, USA), as we previously reported [46,47]. 

### 2.11. Measurement of sE-Cad Levels

The levels of sE-cad were measured using the human soluble E-cadherin ELISA Kit (MyBioSource, San Diego, CA, USA) according to the manufacturer’s instructions. Briefly, the assay is based on sE-cad antibody-sE-cad antigen interactions using an HRP colorimetric detection system to measure sE-cad in samples. Upon addition of the stop solution, the color changes from blue to yellow, and the intensity of the color is measured at 450 nm. By using the provided set of calibration standards, a standard curve of optical density versus sE-cad is generated, allowing measurement of the concentration of sE-cad in the sample. 

### 2.12. Immunodepletion

Immunodepletion (ID) of the conditioned media was carried out according to methods previously described [48] and our recently published reports [35,40,44,45] using sE-cad monoclonal antibody (20 μg/mL) that binds the ectodomain of E-cadherin, DECMA-1 [49]. As a negative control, a human IgG isotype control (hIgG, 20 μg/mL) was used. This ID medium was then carefully removed and analyzed for the presence of sE-cad by using the human soluble E-cadherin ELISA Kit (MyBioSource). 

### 2.13. Western Blotting

Cell lysate samples were collected according to our previous protocols [38,41,45]. Following methods we reported earlier [35,36,42,43], samples were boiled at 95 °C for 10 min in 1 × SDS sample buffer with β-mercaptoethanol, loaded and separated by SDS-PAGE on a 12% gel, then transferred to a nitrocellulose membrane using a Trans-Blot apparatus (Bio-Rad) at 90 V for 1 h. After blocking the membrane in TBST (Tris buffered-saline containing 0.1% *v*/*v* Tween-20) buffer containing 5% nonfat milk for 6 h at 4 °C, the membrane was washed 5× with TBST and then incubated with the MMP9 primary antibody (1:1000) or the α-tubulin antibody (1 µg/mL) in the blocking buffer at 4 °C overnight with gentle shaking on a rotating shaker. After washing 3× with TBST, the membrane was incubated with m-IgG Fc BP-HRP (1:10,000) to detect the MMP9 primary antibody or goat anti-mouse IgG secondary antibody (1:4000 dilution) against α-tubulin primary antibody in the blocking buffer with gentle agitation for 2 h at RT. After washing 3× with TBST, the blots were developed using super signal west pico luminol (chemiluminescence) reagent and imaged with a Bio-Rad molecular imager. 

### 2.14. SiRNA Transfection

Transfections were carried out according to our methods reported previously [41,50]. Control siRNA or MMP9 siRNA (100 nM) were each mixed with Lipofectamine 2000 transfection reagent diluted in Opti-MEM Media (ThermoFisher) and then added to the cells that were allowed to incubate at 37 °C for 12 h, followed by the specific treatments as indicated. Each measurement represents the mean ± S.D. of three-five independent experiments, each performed in triplicate. 

### 2.15. Statistical Analysis

The analysis was performed as we reported earlier [39,40,41,45]. The Mann–Whitney test and a non-parametric Kruskal–Wallis test, followed by Dunn’s multiple comparison test for comparing three or more groups, were performed. 

## 3. Results

### 3.1. MMP9 Levels Increased in the Conditioned Media of A549 and H1299 Cells Treated with Either BDNF, Nicotine, or Epinephrine but Decreased upon Cell Treatment with Non-Selective β-Adrenergic Receptors Blocker, Propranolol

Neurotrophins and MMPs are known to share a complex relationship whereby MMPs are needed to catalyze the extracellular cleavage of secreted pro-neurotrophins, and conversely, the expression and function of MMPs are known to be regulated by neurotrophins [51,52]. BDNF was shown to stimulate MMP9 secretion using human bronchial smooth muscle cells [53], and MMP9 mRNA, protein levels, and activity were upregulated upon stimulation of rat primary cortical neurons with BDNF [54]. MMP9, among other MMPs, is known to be overexpressed in tumorigenesis [55,56]. We have previously found higher levels of proBDNF in the media of A549 cells (p53 wild-type) than in H1299 cells (p53-null) and reported that the ratio of proBDNF/mature BDNF was not affected by MMP2 knockdown but increased in the media of both cell lines upon knockdown of MMP9, suggesting that MMP9 regulates the cytotoxic effects induced by proBDNF in lung cancer cells [35]. Nicotine is known to promote cell invasion and proliferation and induce Epithelial-to-Mesenchymal Transition (EMT) through mechanisms involving downregulation of E-cadherin and increasing expression of mesenchymal markers, including MMP9, in the NSCLC cell line A549 [57]. Activation of β2-ARs by isoproterenol in A549 cells was shown to increase the expression of MMPs, including MMP9, a finding also observed in pancreatic and gastric cancer [28,58,59,60]. Moreover, tumors in stressed animals had a significantly increased expression level of the MMP9 protein [24,28,60].

**Figure 2:**
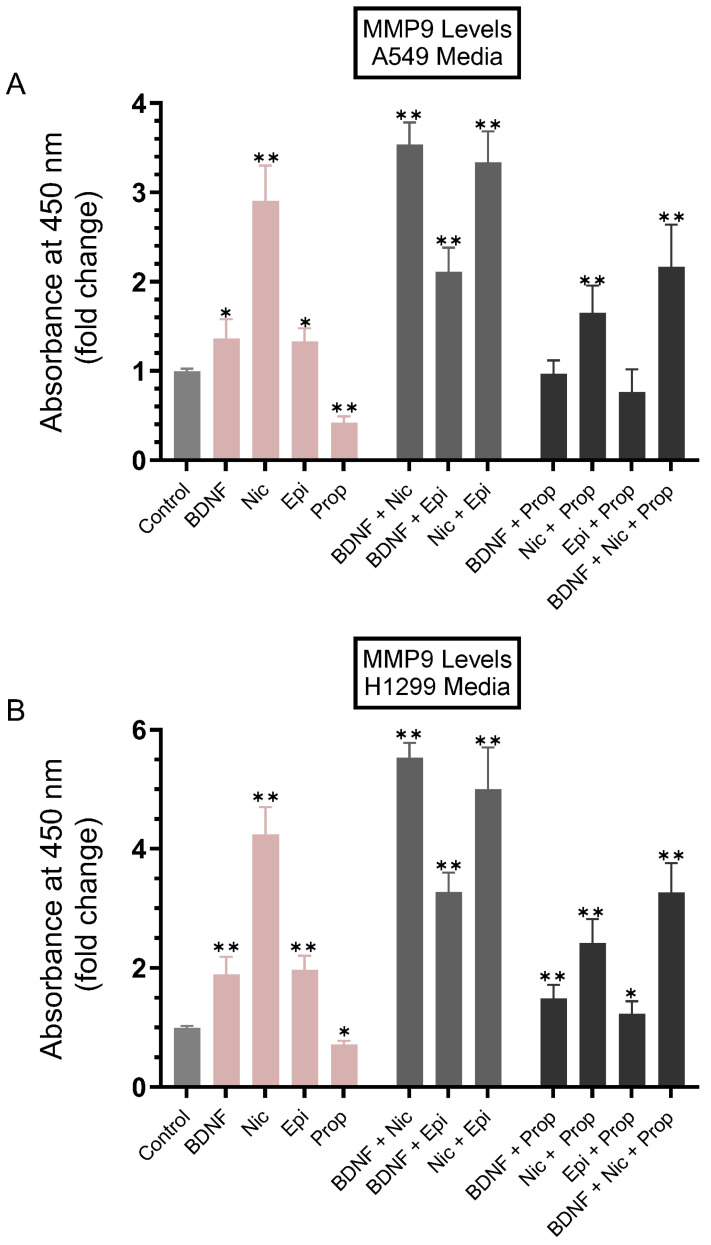
The levels of MMP9 increased in the conditioned media of A549 and H1299 cells treated with either BDNF, nicotine, or epinephrine and decreased upon treatment of cells with propranolol. Cells (0.2 × 10^5^) were grown in 10% FBS-supplemented media for 24 h, then serum starved overnight. The cell monolayers were then treated as indicated for 72 h with BDNF (5 nM), nicotine (Nic, 1 µM), epinephrine (Epi, 100 nM), propranolol (Prop, 1 µM), and in combination. MMP9 levels were quantitated using the media of A549 cells (**A**) and H1299 cells (**B**) (Methods). Data were averaged, normalized, and expressed as fold change relative to untreated cells (Control) using the GraphPad 9.5.1 software (n = 5). Asterisks indicate a statistically significant difference from the corresponding control for each cell line, using the Mann-Whitney test. Please note the differences in scale. For comparing three or more groups, the Kruskal–Wallis test, followed by Dunn’s multiple comparison test, was performed. Absence of asterisks indicates no significance; * *p* < 0.05; ** *p* < 0.0l.

To examine the levels of MMP9 in the media of A549 and H1299 cells treated with BDNF, nicotine, epinephrine, propranolol, and in combination, cells were grown in 10% FBS-supplemented media for 24 h, then serum starved overnight (Figure 2). The cell monolayers were then treated as indicated for 72 h with BDNF, nicotine, epinephrine, propranolol, and in combination (Figure 2). The media of A549 cells and H1299 cells were collected, and the same amount of protein from each sample was used to quantify MMP9 (Methods). Treatment of cells with BDNF increased the levels of MMP9 in the media of A549 cells ~1.35-fold (Figure 2A) and ~1.85-fold in H1299 cell media (Figure 2B), and treatment with epinephrine resulted in a comparable effect to those levels in both cell lines. A more pronounced increase in MMP9 levels in the media was observed upon cell treatment with nicotine (a ~2.90-fold increase for A549 and a ~4.25-fold increase for H1299). Treatment with propranolol decreased the levels of MMP9 in A549 cell media by ~2.35-fold and ~1.40-fold in the media of H1299 cells compared to control untreated cells (Figure 2). Higher levels of MMP9 were found in the media of A549 cells co-treated with BDNF and nicotine (~3.50-fold), BDNF and epinephrine (~2.15-fold), and nicotine and epinephrine (~3.30-fold) (Figure 2A). The increase in MMP9 levels was somewhat higher in the media of H1299 cells (Figure 2B) co-treated with BDNF and nicotine (~5.50-fold), BDNF and epinephrine (~3.25-fold), and nicotine and epinephrine (~5.00-fold) (Figure 2B). Co-treatment with propranolol and either BDNF, nicotine, epinephrine, or a combination of BDNF and nicotine led to reduced levels of MMP9 in the media of both A549 (Figure 2A) and H1299 cells (Figure 2B). 

### 3.2. The Levels of sE-Cad Were Downregulated in the Media of A549 Cells Transfected with MMP9 siRNA

**Figure 3:**
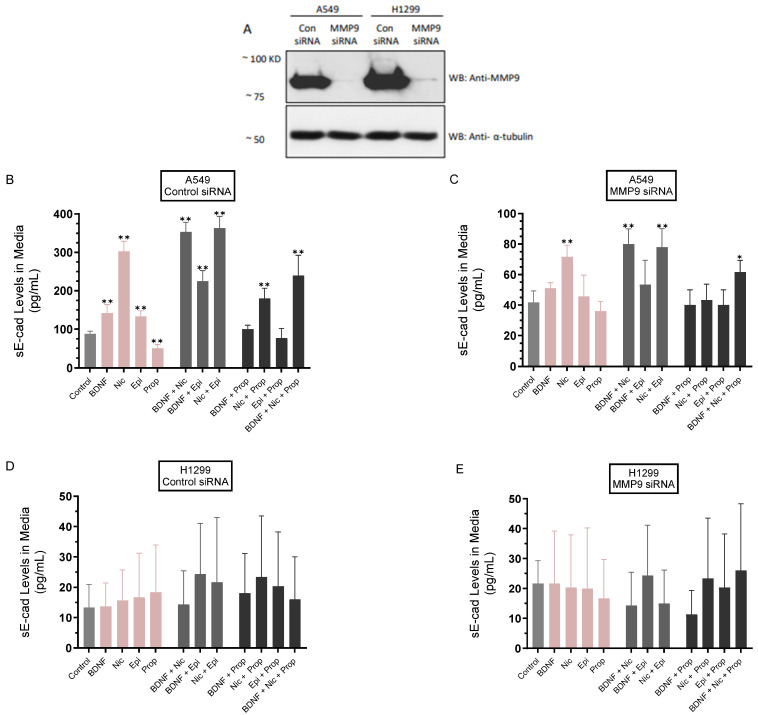
sE-cad levels decreased in the media of A549 cells transfected with MMP9 siRNA. Cells (0.2 × 10^5^) were transfected with the indicated siRNAs (Methods) in the absence or presence of treatment for 72 h with BDNF (5 nM), nicotine (Nic, 1 µM), epinephrine (Epi, 100 nM), propranolol (Prop, 1 µM), and in combination. The same protein concentration of the cell lysates (**A**) was used for Western blotting using antibodies directed against MMP9 or anti α-tubulin. The levels of sE-cad were quantitated in the media of A549 cells (**B**,**C**) and H1299 cells (**D**,**E**) using the same amount of protein (Methods). The data were averaged using the GraphPad 9.5.1 software (n = 5). Asterisks indicate a statistically significant difference from the corresponding control for each cell line using the Mann–Whitney test. Please note the differences in scale. For comparing three or more groups, the Kruskal–Wallis test, followed by Dunn’s multiple comparison test, was performed. Absence of asterisks indicates no significance; * *p* < 0.05; ** *p* < 0.0l.

It was previously shown that curcumin, the main natural polyphenol derived from the rhizomes of Curcuma longa (turmeric) and an active ingredient in traditional herbal remedies, inhibited MMP9 expression, resulting in decreased sE-cad levels in NSCLC cell media and blocking migration and invasion [61]. Our results (Figure 2) showed an increased level of MMP9 in the conditioned media of A549 and H1299 cells treated with either BDNF, nicotine, or epinephrine and decreased MMP9 levels upon treatment of cells with propranolol. To examine the levels of sE-cad under those conditions, cells (Figure 3) were grown in 10% FBS-supplemented media for 24 h, then serum starved overnight. The cells were then transfected with control- or MMP9-siRNAs as described in the Section 2 in the absence or presence of treatment for 72 h with BDNF, nicotine, epinephrine, propranolol, and in combination (Figure 3). The media of A549 cells and H1299 cells were collected, and the same amount of protein from each sample was used to quantitate sE-cad (Methods). 

The levels of sE-cad in the media of A549 cells transfected with control siRNA (Figure 3A,B) and treated with BDNF or epinephrine increased ~1.55-fold compared to control untreated cells. The increase in those levels was more pronounced, ~3.45-fold, upon cell treatment with nicotine under the same conditions, while treatment with propranolol decreased those levels ~1.75-fold (Figure 3B). Co-treatment of A549 cells transfected with control siRNA with nicotine and either BDNF or epinephrine increased sE-cad levels in the media by ~4.00 fold compared to untreated control cells, while that increase was more modest, ~2.55-fold, with cells treated with BDNF and epinephrine under the same conditions (Figure 3B). Compared to treatment without propranolol, co-treatment of A549 cells transfected with control siRNA and propranolol reduced the levels of sE-cad in the media of cells incubated with BDNF (~1.40-fold), nicotine (~1.65-fold), epinephrine (~1.70-fold), and BDNF + nicotine (~1.45-fold) (Figure 3B). 

Transfection of A549 cells with MMP9 siRNA (Figure 3C) resulted in decreased sE-cad levels in the media by ~2.00-fold compared to cells transfected with control siRNA (Figure 3A–C). The levels of sE-cad in the media of A549 cells transfected with MMP9 siRNA (Figure 3A,C) and treated with BDNF or epinephrine increased ~1.20-fold compared to control untreated cells. The increase in those levels was more pronounced, ~1.70-fold, upon cell treatment with nicotine under the same conditions, while treatment with propranolol decreased those levels ~1.15-fold (Figure 3C). Co-treatment of A549 cells transfected with MMP9 siRNA with nicotine and either BDNF or epinephrine increased sE-cad levels in the media ~1.90 fold compared to untreated control cells, while that increase was more modest, ~1.25-fold with cells treated with BDNF and epinephrine under the same conditions (Figure 3C). Compared to treatment without propranolol, co-treatment of A549 cells transfected with MMP9 siRNA and propranolol reduced the levels of sE-cad in the media of cells incubated with BDNF (~1.25-fold), nicotine (~1.55-fold), epinephrine (~1.15-fold), and BDNF + nicotine (~1.35-fold) (Figure 3C). 

A549 cells transfected with MMP9 siRNA and treated with BDNF or epinephrine had decreased levels of sE-cad ~2.80-fold compared to the same treatment of A549 cells transfected with control siRNA (Figure 3B,C). A549 cells transfected with MMP9 siRNA and treated with nicotine resulted in a ~4.20-fold decrease in sE-cad levels in the media compared to control siRNA-transfected A549 cells under the same conditions (Figure 3B,C). Compared to A549 cells treated with control siRNA, treatment of cells transfected with MMP9 siRNA with propranolol resulted in decreased sE-cad levels by ~1.40-fold. Co-treatment of A549 cells transfected with MMP9 siRNA with nicotine and either BDNF or epinephrine decreased sE-cad levels by ~4.40 fold compared to control siRNA transfectants with the same treatments, while that decrease was ~4.25-fold with cells treated with BDNF and epinephrine under the same conditions (Figure 3C). Compared to the same treatments of A549 cells transfected with control siRNA, transfection with MMP9 siRNA led to decreased levels of sE-cad in the media of cells incubated with BDNF + propranolol (~2.50-fold), nicotine + propranolol (~4.20-fold), epinephrine + propranolol (~1.90-fold), and BDNF + nicotine (~4.15-fold) (Figure 3B,C). In contrast to A549 cells, no sE-cad was detected in the media of H1299 cells under all conditions tested (Figure 3A,D,E). The lack of E-cadherin expression in H1299 cells as compared to A549 cells is consistent with previous studies showing that in H1299 cells, E-cadherin is not detectable [62,63].

### 3.3. Immunodepletion of sE-Cad Decreased Viability and Increased Apoptosis of A549 Cells

**Figure 4:**
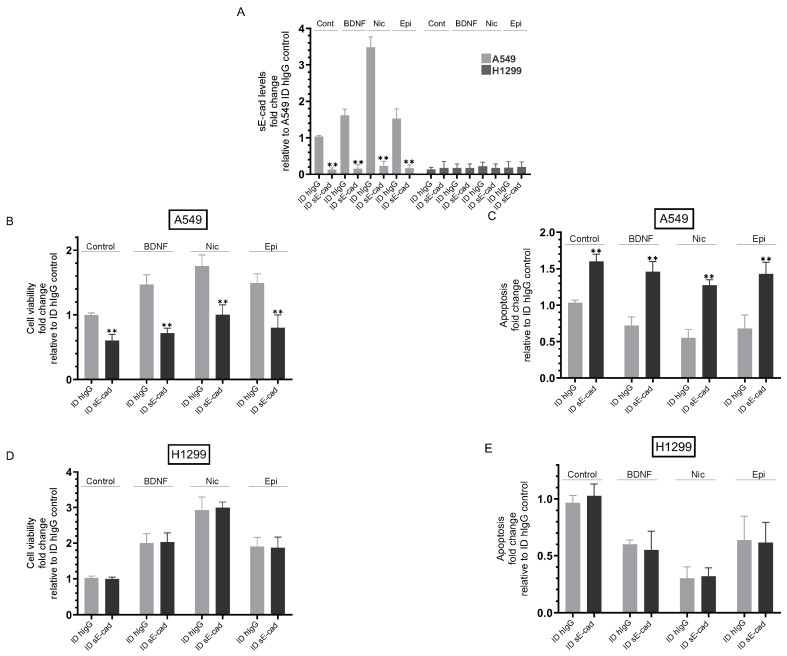
Immunodepletion of sE-cad led to decreased viability and increased apoptosis in A549 cells. Media immunodepleted (ID) of sE-cad was prepared by first growing cells (0.2 × 10^5^) in 10% FBS-supplemented media for 24 h. The cells were then incubated in serum-free media overnight and either untreated (Control) or treated for 72 h with BDNF (5 nM), nicotine (Nic, 1 µM), or epinephrine (Epi, 100 nM). The media was then collected and depleted with either hIgG (ID hIgG) or sE-cad (ID sE-cad) (Methods, (**A**)). The viability and apoptosis of A549 (**B**,**C**) and H1299 (**D**,**E**) cells were next assessed (Methods). Data were averaged, normalized, and expressed as fold change relative to ID hIgG control untreated cells using the GraphPad 9.5.1 software (n = 5). Asterisks indicate a statistically significant difference between each treatment ID from sE-cad relative to the same treatment ID using hIgG, ** *p* < 0.0l. Please note the differences in scale. Asterisks indicate a statistically significant difference from the corresponding control for each cell line, using the Mann–Whitney test. For comparing three or more groups, the Kruskal–Wallis test, followed by Dunn’s multiple comparison test, was performed. The absence of asterisks indicates no significance; ** *p* < 0.0l.

Previous studies have shown that sE-cad acts as an anti-apoptotic factor [7,11]. To examine the effects of sE-cad on cell viability and apoptosis under our conditions, cells were incubated with media immunodepleted using either hIgG with no relevant specificity to a target antigen or a sE-cad monoclonal antibody that binds the ectodomain of E-cadherin from cells either untreated (Control) or treated for 72 h with BDNF, nicotine, or epinephrine as described in the Figure 4 legend and in the Section 2. To test cell viability or apoptosis, cells were seeded in 96-well plates in 10% FBS-supplemented media for 24 h, then incubated in serum-free media for 12 h. The cells were then incubated for 72 h with media from the different treatments immunodepleted using either hIgG or sE-cad antibodies, with the media containing the specific components in the various treatments replaced every 12 h. The viability and apoptosis of A549 and H1299 cells were assessed as described in the Section 2.

Relative to hIgG immunodepleted media, immunodepletion of sE-cad from the media (Figure 4A) of untreated A549 cells resulted in a ~1.65-fold decrease in viability (Figure 4B) and an increase in apoptosis (Figure 4C). Using hIgG immunodepleted media, A549 cell treatment with BDNF increased viability ~1.45-fold and decreased apoptosis ~1.35-fold (Figure 4B,C) relative to control cells. Compared to treatment using hIgG immunodepleted media of BDNF-treated A549 cells, immunodepletion of sE-cad from the media resulted in a ~2.00-fold decrease in viability and an increase in apoptosis (Figure 4B,C). Using hIgG immunodepleted media from A549 cells treated with nicotine increased viability ~1.75-fold and decreased apoptosis ~1.80-fold (Figure 4B,C). Immunodepletion of sE-cad from the media of A549 cells treated with nicotine decreased viability ~1.75-fold (Figure 4B) and increased apoptosis ~2.30-fold (Figure 4C) compared to hIgG immunodepleted media from nicotine-treated A549 cells. Using hIgG immunodepleted media from A549 cells treated with epinephrine led to a ~1.50-fold increase in cell viability, while apoptosis decreased ~1.45-fold under the same conditions (Figure 4B,C). Similar to the results obtained for A549 cells treated with either BDNF or nicotine, immunodepletion of sE-cad from the media of epinephrine-treated A549 cells decreased cell viability by ~1.85-fold and increased apoptosis by ~2.00-fold (Figure 4B,C) relative to the same treatment using hIgG immunodepleted media. Unlike the results obtained when using A549 cells, no difference was found upon immunodepletion using either hIgG or sE-cad antibodies on H1299 cell viability of apoptosis under all conditions tested (Figure 4D,E), results consistent with previous reports showing that H1299 cells are negative for E-cadherin [62,63]. Treatment of H1299 cells with BDNF or epinephrine increased cell viability ~2.00-fold (Figure 4D) and decreased apoptosis ~1.65-fold (Figure 4E), while nicotine treatment increased viability ~2.95-fold (Figure 4D) and decreased apoptosis ~3.35-fold (Figure 4E). 

### 3.4. The Activities of EGFR and IGF-1R Were Blocked by Immunodepletion of sE-Cad from the Media of A549 Cells

E-cadherin was detected in early-stage ovarian carcinoma [7,10] and functioned to increase cell survival and proliferation by inducing EGFR dimerization and activation in a ligand-independent manner [5,7]. The extracellular domain of E-cadherin, sE-cad, generated by cleavage and shedding, is known to physically interact with EGFR, thus acting as a soluble growth factor [11,13]. It has also been demonstrated that all four members of the EGFR family (HER1-4) are activated by sE-cad [11,13]. A reciprocal relationship has been suggested between EGFR and sE-cad since EGFR activation is known to result in MMP- and ADAM-dependent generation of sE-cad, suggesting a feedback mechanism whereby activation of EGFR leads to the production of sE-cad, which can then further increase EGFR signaling promoting oncogenic proliferation [9,11,12,13,49]. Stronger effects have been found for sE-cad as compared to EGF, the classical ligand of EGFR, in increasing cancer cell proliferation and metastasis [13]. The IGF-1R, known to play an important role in regulating cell proliferation and tumor progression, has also been shown to form a complex with and be activated by sE-cad [12]. We have previously shown that NSCLC cell treatment with epinephrine or nicotine resulted in activation of EGFR and IGF-1R, an effect opposite to that found by cell treatment with propranolol [37].

**Figure 5:**
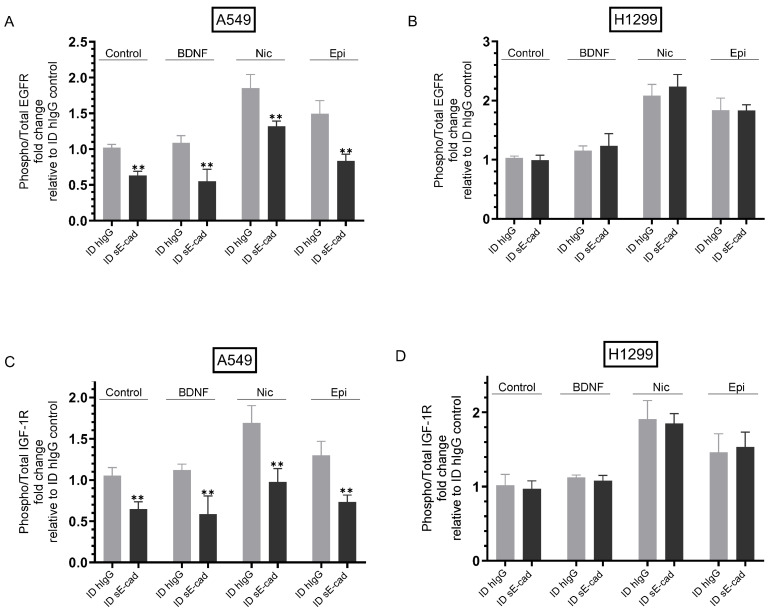
Immunodepletion of sE-cad inhibited activation of EGFR and IGF-1R in A549 cells. Cells were incubated in serum-free media for 12 h, then treated for 72 h with 300 μL of media (0.5 μg/μL) immunodepleted using either hIgG (ID hIgG) or sE-cad (ID sE-cad) (Methods) prepared from cells either untreated (Control) or treated with BDNF (5 nM), nicotine (Nic, 1 µM), or epinephrine (Epi, 100 nM), as described in the Figure 4 legend. The phospho/total EGFR assay (**A**,**B**) and the phospho/total IGF-1R assay (**C**,**D**) were then carried out (Methods). Data were expressed as fold change relative to ID hIgG control untreated cells using the GraphPad 9.5.1 software (n = 5). Asterisks indicate a statistically significant difference between each treatment ID from sE-cad relative to the same treatment ID using hIgG, ** *p* < 0.0l. Please note the differences in scale. Asterisks indicate a statistically significant difference from the corresponding control for each cell line, using the Mann–Whitney test. For comparing three or more groups, the Kruskal–Wallis test, followed by Dunn’s multiple comparison test, was performed. The absence of asterisks indicates no significance; ** *p* < 0.0l.

Our results (Figure 4) showed that immunodepletion of sE-cad led to decreased viability and increased apoptosis in A549 cells. To examine the possible involvement of EGFR and IGF-1R in this function, cells were seeded in 10% FBS-supplemented media overnight and then incubated in serum-free media for 12 h. The cells were then treated with hIgG- or sE-cad-immunodepleted media prepared from cells either untreated (Control) or treated with BDNF, nicotine, or epinephrine as described in the Figure 4 legend and in the Section 2 for 72 h, with the media containing the specific components in the various treatments replaced every 12 h. The phospho/total EGFR assay and the phospho/total IGF-1R assay were then carried out as described in the Section 2 (Figure 5). 

Treatment of control A549 cells with media immunodepleted (ID) of sE-cad decreased EGFR activation ~1.70-fold (Figure 5A). While minimal activation of EGFR was found upon treatment of A549 cells with BDNF, treatment with nicotine or epinephrine resulted in ~1.85-fold and ~1.50-fold EGFR activation, respectively (Figure 5A). Compared to the corresponding ID hIgG media, EGFR activation was decreased in cells treated with BDNF + ID sE-cad (~1.75-fold), nicotine + ID sE-cad (~1.45-fold), and epinephrine + ID sE-cad (~1.80-fold) (Figure 5A). While little to no effect was found upon H1299 cell treatment with BDNF on the activation of EGFR, treatment with nicotine or epinephrine increased EGFR activation ~2.00-fold and ~1.85-fold, respectively (Figure 5B). Consistent with the low sE-cad levels detected in the media of H1299 cells (Figure 3), no difference in EGFR activation was observed in H1299 cells treated with hIgG- or sE-cad-ID media prepared from control untreated cells or cells treated with BDNF, nicotine, or epinephrine under all conditions tested (Figure 5B). 

Similar trends were observed for both cell lines tested for activation of IGF-1R under the same conditions (Figure 5C,D). Control A549 cells incubated with media ID of sE-cad decreased IGF-1R activation ~1.55-fold relative to cells incubated with ID hIgG media (Figure 5C). While little to no activation of IGF-1R was found upon treatment of A549 cells with BDNF, treatment with nicotine or epinephrine resulted in ~1.60-fold and ~1.30-fold IGF-1R activation, respectively (Figure 5C). Compared to treatments using ID hIgG media, IGF-1R activation was diminished in cells treated with BDNF + ID sE-cad (~1.90-fold), nicotine + ID sE-cad (~1.55-fold), and epinephrine + sE-cad (~1.75-fold) (Figure 5C). While little to no effect was found upon H1299 cell treatment with BDNF on the activation of IGF-1R, treatment with nicotine or epinephrine increased IGF-1R activation ~1.90-fold and ~1.45-fold, respectively (Figure 5D). Since H1299 cells are negative for E-cadherin [62,63], no effects on IGF-1R activation were found in cells treated with hIgG- or sE-cad-ID media prepared from control untreated cells or cells treated with BDNF, nicotine, or epinephrine under all conditions tested (Figure 5D). 

### 3.5. Immunodepletion of sE-Cad Led to Decreased PI3K and ERK1/2 Activities and Enhanced p53 Activation in A549 Cells

**Figure 6:**
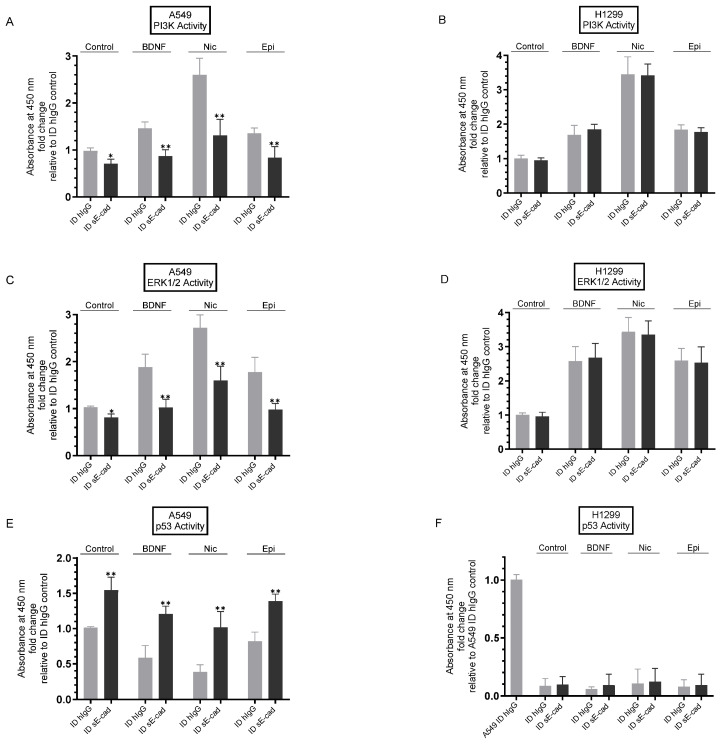
Immunodepletion of sE-cad resulted in decreased PI3K and ERK1/2 activities and increased activation of p53 in A549 cells. Cells were incubated in serum-free media for 12 h, then treated for 72 h with 300 μL of media (0.5 μg/μL) immunodepleted using either hIgG (ID hIgG) or sE-cad (ID sE-cad) (Methods) prepared from cells either untreated (Control) or treated with BDNF (5 nM), nicotine (Nic, 1 µM), or epinephrine (Epi, 100 nM), as described in the Figure 4 legend. The media containing the specific components in the various treatments were replaced every 12 h. The PI3K activity (**A**,**B**), the ERK1/2 activity (**C**,**D**), and the p53 activity (**E**,**F**) were then carried out (Methods). Data were expressed as fold change relative to ID hIgG control untreated cells (**A**–**E**) or relative to A549 ID hIgG control (**F**) using the GraphPad 9.5.1 software (n = 5). Asterisks indicate a statistically significant difference between each treatment ID from sE-cad and the same treatment ID using hIgG. Asterisks indicate a statistically significant difference from the corresponding control for each cell line, using the Mann–Whitney test. Please note the differences in scale. For comparing three or more groups, the Kruskal–Wallis test, followed by Dunn’s multiple comparison test, was performed. Absence of asterisks indicates no significance; * *p* < 0.05; ** *p* < 0.0l.

Several reports have shown that sE-cad inhibits apoptosis and has an important function in cancer progression via activation of several signaling cascades linked to tumor progression, such as the EGFR and IGF-1R signaling pathways, and consequent activation of ERK1/2 and PI3K signaling [5,7,8,13]. We reported earlier that the activities of PI3K and AKT were upregulated by NSCLC cell treatment with nicotine or BDNF and downregulated by cell treatment with inhibitors against EGFR or IGF-1R and by propranolol [37]. We, therefore, set out to examine the effect of sE-cad immunodepletion on PI3K, ERK1/2, and p53 activities (Figure 6). 

Cells were seeded in 96-well plates in 10% FBS-supplemented media overnight, then incubated in serum-free media for 12 h. The cells were then treated for 72 h with hIgG- or sE-cad-immunodepleted media prepared from cells either untreated (Control) or treated with BDNF, nicotine, or epinephrine as described in the Figure 4 legend and in the Section 2. The PI3K activity (Figure 6A,B), the ERK1/2 activity (Figure 6C,D), and the p53 activity (Figure 6E,F) were then carried out as described in the Section 2. 

Compared to control cell incubation with ID hIgG media, A549 cell treatment with ID sE-cad media resulted in a ~1.40-fold decrease in the activity of PI3K (Figure 6A), while no change in PI3K activation was found when using H1299 cells under the same conditions (Figure 6B). The activity of PI3K in A549 cells incubated with ID hIgG media from the different treatments increased as follows: ~1.40-fold with BDNF, ~2.50-fold with nicotine, and ~1.35-fold with epinephrine (Figure 6A). Treatment of cells with ID sE-cad media from the same treatments decreased the activity of PI3K relative to treatment using ID hIgG media as follows: [BDNF + ID sE-cad (~1.65-fold), nicotine + ID sE-cad (~1.90-fold), and epinephrine + sE-cad (~1.65-fold)] (Figure 6A). H1299 cell treatment increased the activity of PI3K ~1.65-fold, ~3.45-fold, and ~1.85-fold with BDNF, nicotine, or epinephrine, respectively, whether the cells were incubated with ID hIgG or ID sE-cad media (Figure 6B). The same treatments measuring the activity of ERK1/2 resulted in similar trends for both cell lines (Figure 6C,D). Compared to incubation with control ID hIgG media, A549 cell incubation with ID sE-cad media resulted in a ~1.25-fold decrease in the activity of ERK1/2 (Figure 6C), while no change was observed for H1299 cells under the same conditions (Figure 6D). The activity of ERK1/2 in A549 cells incubated with ID hIgG media from the different treatments increased as follows: ~1.85-fold with BDNF, ~2.70-fold with nicotine, and ~1.75-fold with epinephrine (Figure 6C). Incubation of A549 cells with ID sE-cad media from the same treatments diminished the activity of ERK1/2 relative to treatment with ID hIgG media as follows: [BDNF + ID sE-cad (~1.80-fold), nicotine + ID sE-cad (~1.60-fold), and epinephrine + sE-cad (~1.80-fold)] (Figure 6C). Using H1299 cells, the activity of ERK1/2 increased ~2.55-fold, ~3.45-fold, and ~2.60-fold with BDNF, nicotine, or epinephrine, respectively, whether the cells were incubated with ID hIgG or ID sE-cad media (Figure 6D). 

Opposite trends to those found for the activity of PI3K (Figure 6A,B) or ERK1/2 (Figure 6C,D) were observed for p53 activation (Figure 6 E,F). A549 cell treatment with ID sE-cad media resulted in a ~1.55-fold increase in the activity of p53 (Figure 6E) relative to control hIgG media. The p53 activity in A549 cells incubated with ID hIgG media from the different treatments decreased as follows: ~1.70-fold with BDNF, ~2.65-fold with nicotine, and ~1.20-fold with epinephrine (Figure 6E). Incubation of cells with ID sE-cad media from the same treatments increased the activity of p53 relative to treatment with ID hIgG media as follows: [BDNF + ID sE-cad (~2.00-fold), nicotine + ID sE-cad (~2.70-fold), and epinephrine + sE-cad (~1.70-fold)] (Figure 6E). No p53 activity was detected in H1299 cells under all conditions tested (Figure 6F), a finding not surprising since H1299 cells are known to have a p53-null genotype due to a biallelic deletion of the *TP53* gene [64].

## 4. Discussion

Dysfunction of E-cadherin is known to lead to increased tumor metastasis capacity [7,14]. Membrane-bound E-cadherin is known to play a critical role in maintaining cell-cell adhesion in epithelial cells and negatively regulates tumor survival and growth in the lung [8,63]. Cell–cell adhesions formed by the protein are thought to occur via E-cadherin calcium-dependent homophilic interactions, and downregulation of E-cadherin is known to lead to reduced cell adhesion [6,8,14,65]. Opposite functions have been reported for sE-cad and full-length E-cadherin (Figure 1) [7,8,11,12,13,14,63,65]. Migration and invasion were reported to be caused by sE-cad in NSCLC cells, and cell treatment with curcumin blocked sE-cad levels by down-regulating expression of MMP9 [61].

In this study, we found increased MMP9 levels in the conditioned media of A549 and H1299 cells treated with either BDNF, nicotine, or epinephrine that were decreased upon cell treatment with the non-selective β-adrenergic receptor blocker propranolol (Figure 2). Higher levels of MMP9 were found in the media of A549 and H1299 cells co-treated with BDNF and nicotine, BDNF and epinephrine, and nicotine and epinephrine (Figure 2). Co-treatment with propranolol and BDNF, nicotine, epinephrine, and a combination of BDNF and nicotine led to reduced levels of MMP9 in the media of both A549 and H1299 cells (Figure 2). 

The levels of sE-cad were downregulated in the media of A549 cells transfected with MMP9 siRNA (Figure 3). These results are consistent with previous findings showing that curcumin inhibits MMP9 expression, resulting in decreased sE-cad levels in NSCLC cell media and blocking migration and invasion [61]. The levels of sE-cad in the media of A549 cells transfected with control siRNA (Figure 3A,B) and treated with BDNF or epinephrine increased compared to control untreated cells, while treatment with propranolol decreased those levels (Figure 3B). The increase in sE-cad levels was more pronounced upon A549 cell treatment with nicotine under the same conditions (Figure 3B). Nicotine was previously found to promote anchorage-independent growth in NSCLCs and induce changes in gene expression consistent with EMT, in part by reducing E-cadherin expression [66]. Nicotine was also found to upregulate MMPs [24,26,67]. Transfection of A549 cells with MMP9 siRNA resulted in decreased sE-cad levels in the media (Figure 3A–C). A549 cells transfected with MMP9 siRNA and treated with BDNF, nicotine, or epinephrine had decreased levels of sE-cad compared to the same treatment of A549 cells transfected with control siRNA (Figure 3B,C). In contrast to the findings with A549 cells, no sE-cad was detected in the media of H1299 cells under all conditions tested (Figure 3A,D,E), an observation consistent with earlier reports showing that in H1299 cells, E-cadherin is not detectable [62,63].

Immunodepletion of sE-cad, using a monoclonal antibody that is able to bind the ectodomain of E-cadherin [49], from the media of A549 cells untreated or treated with either BDNF, nicotine, or epinephrine decreased cell viability and increased apoptosis (Figure 4), a finding consistent with previous reports showing that sE-cad acts as an anti-apoptotic factor [7,11]. Treatment of H1299 cells with BDNF, nicotine, or epinephrine increased cell viability (Figure 4D) and decreased apoptosis (Figure 4E); however, unlike the results obtained when using A549 cells, no effect was found upon immunodepletion of sE-cad on H1299 cell viability or apoptosis under all conditions tested (Figure 4D,E), results in accord with previous studies showing that H1299 cells are negative for E-cadherin [62,63].

**Figure 7:**
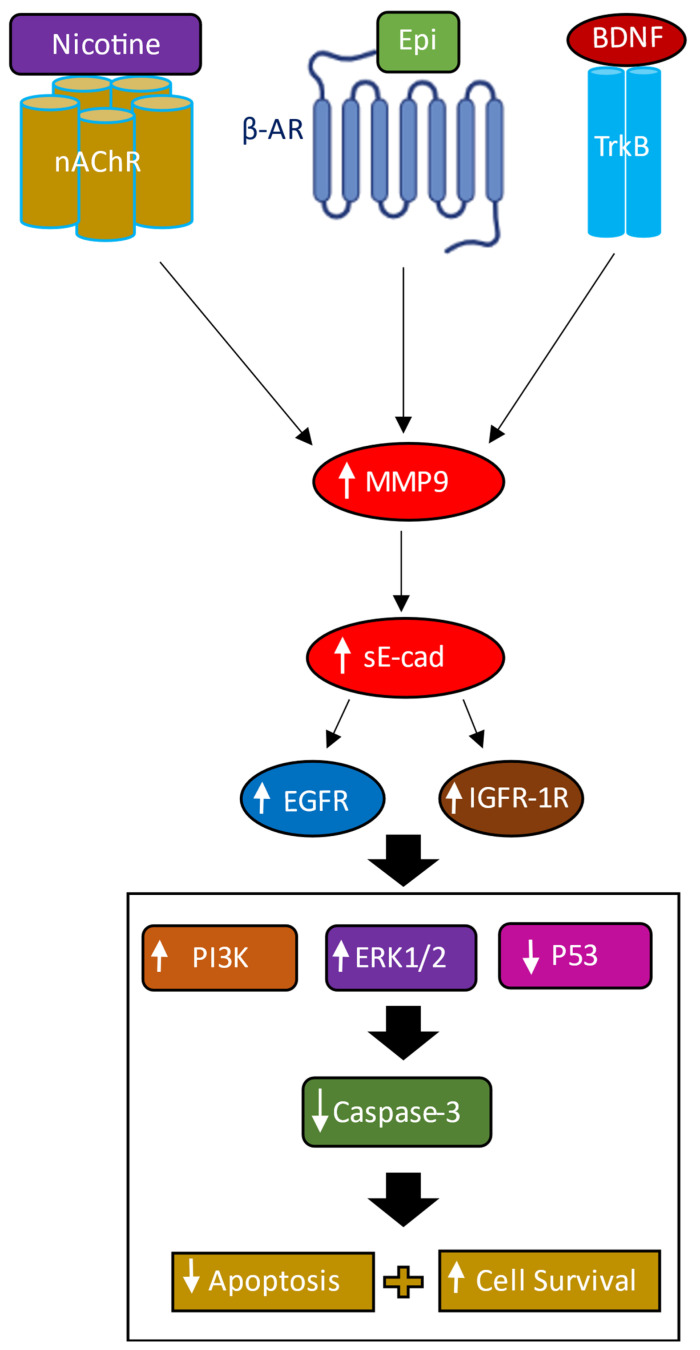
Summary of the findings from this study using A549 cells. The levels of MMP9 increased upon cell treatment with nicotine, epinephrine (Epi), or BDNF. Increased MMP9 levels lead to an increase in the levels of sE-cad in the media, increasing activation of EGFR and IGF-1R, enhancing PI3K and ERK1/2 signaling, and inhibiting p53 activity, resulting in decreased apoptosis and increased cell survival.

Stimulation of EGFR has been shown to increase activation of MMPs and ADAMs, further promoting production of sE-cad, suggesting the existence of a positive feedback loop between EGFR activation and sE-cad production, increasing cancer cell proliferation [8]. Similarly, increased IGF-1R phosphorylation and activation upregulated MMP and ADAM expression and increased in parallel with increased sE-cad, activating the downstream ERK1/2 and PI3K/AKT signaling pathways, thus forming a positive feedback loop to promote cancer development [8,12,13]. These studies have shown that sE-cad-mediated activation of the EGFR/IGF-1R signaling pathways can contribute to tumor development [8,11,12,13]. In this study, we found that the activities of EGFR and IGF-1R (Figure 5) were blocked by immunodepletion of sE-cad from the media of A549 cells untreated or treated with BDNF, nicotine, or epinephrine, while no effects were found when using H1299 cells since they are negative for E-cadherin [62,63]. Immunodepletion of sE-cad also resulted in decreased PI3K and ERK1/2 activities and enhanced p53 activation in A549 cells, either untreated or treated with BDNF, nicotine, or epinephrine (Figure 6). Our findings from this study are summarized in Figure 7. 

Cadherins are known to be regulators of normal tissue development with clinical translational potential [6,7,8,14,30,65,68]. Several studies have shown abnormal levels of sE-cad in tumorigenesis and metastasis [10,11,61]. Therefore, a better understanding of the mechanisms regulating sE-cad levels is an important strategy that may lay the groundwork for future clinical cancer applications. In this study, we shed light on a molecular mechanism whereby the levels of sE-cad in human lung cancer cell media are enhanced by nicotine, BDNF, and/or β-AR ligands, as well as the intracellular signaling mechanisms employed by sE-cad to increase lung cancer cell survival. This work might be clinically significant because targeting these mechanisms can lead to potential therapeutic applications in the treatment of lung cancer.

This study uses cell lines, which are widely considered an in vitro model system in basic cancer research and drug design. However, there is a limitation to translating knowledge from this research of the cellular mechanisms, both regulating sE-cad levels and employed by sE-cad in regulating cell survival, to fully unravel the operative mechanisms in patient tumors. Therefore, translational research targeting sE-cad as a therapeutic target is a promising strategy for cancer treatment with a high potential for driving progress in the field of NSCLC. 

## 5. Conclusions

In this study, our data show that increasing the levels of MMP9 upon A549 cell treatment with nicotine, epinephrine, or BDNF increased sE-cad levels in the media, enhanced EGFR and IGF-1R signaling, led to activation of PI3K and ERK1/2, and inhibited p53 activity, blocking apoptosis and increasing cell survival. Our results from this study provide new insights into a molecular mechanism for the regulation of sE-cad levels in human lung cancer cell media by nicotine, BDNF, and/or β-AR ligands, which can likely offer novel therapeutic opportunities aimed at targeting this regulatory network. 

## Data Availability

Not applicable.
